# A linear oscillator model predicts dynamic temporal attention and pupillary entrainment to rhythmic patterns

**DOI:** 10.16910/jemr.11.2.12

**Published:** 2018-11-20

**Authors:** Lauren K. Fink, Brian K. Hurley, Joy J. Geng, Petr Janata

**Affiliations:** University of California, Davis, USA

**Keywords:** Pupil, attention, entrainment, rhythm, music, modeling, amplitude envelope, psychophysics

## Abstract

Rhythm is a ubiquitous feature of music that induces specific neural modes of processing. In this paper, we assess the potential of a stimulus-driven linear oscillator model (57) to predict dynamic attention to complex musical rhythms on an instant-by-instant basis. We use perceptual thresholds and pupillometry as attentional indices against which to test our model predictions. During a deviance detection task, participants listened to continuously looping, multiinstrument, rhythmic patterns, while being eye-tracked. Their task was to respond anytime they heard an increase in intensity (dB SPL). An adaptive thresholding algorithm adjusted deviant intensity at multiple probed temporal locations throughout each rhythmic stimulus. The oscillator model predicted participants’ perceptual thresholds for detecting deviants at probed locations, with a low temporal salience prediction corresponding to a high perceptual threshold and vice versa. A pupil dilation response was observed for all deviants. Notably, the pupil dilated even when participants did not report hearing a deviant. Maximum pupil size and resonator model output were significant predictors of whether a deviant was detected or missed on any given trial. Besides the evoked pupillary response to deviants, we also assessed the continuous pupillary signal to the rhythmic patterns. The pupil exhibited entrainment at prominent periodicities present in the stimuli and followed each of the different rhythmic patterns in a unique way. Overall, these results replicate previous studies using the linear oscillator model to predict dynamic attention to complex auditory scenes and extend the utility of the model to the prediction of neurophysiological signals, in this case the pupillary time course; however, we note that the amplitude envelope of the acoustic patterns may serve as a similarly useful predictor. To our knowledge, this is the first paper to show entrainment of pupil dynamics by demonstrating a phase relationship between musical stimuli and the pupillary signal.

## Introduction

Though diverse forms of music exist across the globe, all music shares the property of evolving through time. While certain scales, modes, meters, or timbres may be more or less prevalent depending on the culture in question, the use of time to organize sound is universal. Therefore, rhythm, one of the most basic elements of music, provides an excellent scientific starting point to begin to question and characterize the neural mechanisms underlying music-induced changes in motor behavior and attentional state. To remain consistent with previous literature, here rhythm is defined as patterns of duration, timing, and stress in the amplitude envelope of an auditory signal (a physical property), whereas meter is a perceptual phenomenon that tends to include the pulse (beat or tactus) frequency perceived in a rhythmic sequence, as well as slower and faster integer-related frequencies (1).

Previous studies have shown that the presence of meter affects attention and motor behavior. For instance, perceptual sensitivity is enhanced and reaction times are decreased when targets occur in phase with an on-going metric periodicity (2, 3, 4). Interestingly, this facilitation via auditory regularity is observed not only for auditory targets, including speech (5), but also for visual targets (6, 7, 8, 9, 10, 11, 12). One promising theory that accounts for these results is Dynamic Attending Theory (DAT) (13, 14).

### Dynamic Attending Theory

Dynamic Attending Theory (DAT) posits that the neural mechanisms of attention are susceptible to entrainment by an external stimulus, allowing for temporal predictions and therefore attention and motor coordination to specific time points (13, 14, 15). For any given stimulus, the periodicities with the most energy will capture attention most strongly. Neurobiologically, the proposed mechanism is entrainment of neuronal membrane potential of, for example, neurons in primary auditory cortex (in the case of auditory entrainment), to the external stimulus. These phase-locked fluctuations in membrane potential alter the probability of firing action potentials at any given point in time (see (16) for a review).

Similarly, the recent Active Sensing Hypothesis (16, 17, 18) proposes that perception occurs actively via motor sampling routines, that neural oscillations serve to selectively enhance or suppress input, cross-modally, and that cortical entrainment is, in and of itself, a mechanism of attentional selection. Higher frequency oscillations can become nested within lower frequency ones via phase-phase coupling, phase-amplitude coupling, or amplitude-amplitude coupling, allowing for processing of different stimulus attributes in parallel (19, 20). (21) have connected the ideas of Active Sensing to those of DAT by highlighting the critical role of low frequency neural oscillations. In summary, DAT and Active Sensing are not incompatible, as outlined in (22).

Interestingly, studies of neural entrainment are typically separate from those investigating sensorimotor synchronization, defined as spontaneous synchronization of one’s motor effectors with an external rhythm (23). However, a recent study confirms that the amplitude of neural entrainment at the beat frequency explains variability in sensorimotor synchronization accuracy, as well as temporal prediction capabilities (24). Although motor entrainment, typically referred to as sensorimotor synchronization, is not explicitly mentioned as a mechanism of DAT, the role of the motor system in shaping perception is discussed in many DAT papers (25, 26, 27, 28, 29) and is a core tenet of the Active Sensing Hypothesis (22).

In this paper, we test whether a computational model of Dynamic Attending Theory can predict attentional fluctuations to rhythmic patterns. We also attempt to bridge the gap between motor and cortical entrainment by investigating coupling of pupil dynamics to musical stimuli. We consider the pupil both a motor behavior and an overt index of attention, which we discuss in more detail below.

### Sensori(oculo)motor couplin

Though most sensorimotor synchronization research has focused on large-scale motor effectors, the auditory system also seems to have a tight relationship with the ocular motor system. For instance, (30) show that eye movements can synchronize with a moving acoustic target whether it is real or imagined, in light or in darkness. With regard to rhythm, a recent paper by (31) suggests that the tempo of rhythmic auditory stimuli modulates both fixation durations and inter-saccade-intervals: rhythms with faster tempi result in shorter fixations and inter-saccade-intervals and vice versa. These results seem to fit with those observed in audiovisual illusions, which illustrate the ability of auditory stimuli to influence visual perception and even enhance visual discrimination (32, 33). Such cross-modal influencing of perception also occurs when participants are asked to engage in purely imaginary situations (34).

Though most studies have focused on eyeball movements, some (outlined below) have begun to analyze the effect of auditory stimuli on pupil dilation. Such an approach holds particular promise, as changes in pupil size reflect sub-second changes in attentional state related to locus coeruleus-mediated noradrenergic (LC-NE) functioning (35, 36, 37). The LC-NE system plays a critical role in sensory processing, attentional regulation, and memory consolidation. Its activity is time-locked to theta oscillations in hippocampal CA1, and is theorized to be capable of phase-resetting forebrain gamma band fluctuations, which are similarly implicated in a broad range of cognitive processes (38).

In the visual domain, the pupil can dynamically follow the frequency of an attended luminance flicker and index the allocation of visual attention (39), as well as the spread of attention, whether cued endogenously or exogenously (40). However, such a pupillary entrainment effect has never been studied in the auditory domain. Theoretically though, pupil dilation should be susceptible to auditory entrainment, like other autonomic responses, such as respiration, heart rate, and blood pressure, which can become entrained to slow periodicities present in music (see (41), for a recent review on autonomic entrainment).

In the context of audition, the pupil seems to be a reliable index of neuronal auditory cortex activity and behavioral sensory sensitivity. (42) simultaneously recorded neurons in auditory cortex, medial geniculate (MG), and hippocampal CA1 in conjunction with pupil size, while mice detected auditory targets embedded in noise. They found that pupil diameter was tightly related to both ripple activity in CA1 (in a 180 degree antiphase relationship) and neuronal membrane fluctuations in auditory cortex. Slow rhythmic activity and high membrane potential variability were observed in conjunction with constricted pupils, while high frequency activity and high membrane potential variability were observed with largely dilated pupils. At intermediate levels of pupil dilation, the membrane was hyperpolarized and the variance in its potential was decreased. The same inverted U relationship was observed for MG neurons as well. Crucially, in the behavioral task, (42) found that the decrease in membrane potential variance at intermediate pre-stimulus pupil sizes predicted the best performance on the task. Variability of membrane potential was smallest on detected trials (intermediate pupil size), largest on false alarm trials (large pupil size), and intermediate on miss trials (small pupil size). Though this study was performed on mice, it provides compelling neurophysiological evidence for using pupil size as an index of auditory processing.

The same inverted U relationship between pupil size and task performance has been observed in humans during a standard auditory oddball task. For instance, (43) showed that baseline pupil diameter predicts both reaction time and P300 amplitude in an inverted U fashion on an individual trial basis. Additionally, (43) found that baseline pupil diameter is negatively correlated with the phasic pupillary response elicited by deviants. Because of the well-established relationship between tonic neuronal activity in locus coeruleus and pupil diameter, it is theorized that both the P300 amplitude and pupil diameter index locus coeruleus-norepinephrine activity (35, 37, 43, 44, 45).

Moving towards musical stimuli, pupil size has been found to be larger for: more arousing stimuli (46, 47), well-liked stimuli (48), more familiar stimuli (47), psychologically and physically salient auditory targets (49, 50, 51), more perceptually stable auditory stimuli (52), and chill-evoking musical passages (53). A particularly relevant paper by (54) showed that the pupil responded to unattended omissions in on-going rhythmic patterns when the omissions coincided with strong metrical beats but not weak ones, suggesting that the pupil is sensitive to internally generated hierarchical models of musical meter. While Damsma and van Rijn’s analysis of difference waves was informative, the continuous time series of pupil size may provide additional dynamic insights into complex auditory processing.

Of particular note, (55) demonstrated a relationship between attention and the time course of the pupillary signal while listening to music. To do this, they had participants listen to 30-sec clips of classical music while being eye-tracked. In the first phase of the experiment, participants listened to each clip individually (diotic presentation); in the second phase participants were presented with two different clips at once (dichotic presentation) and instructed to attend to one or the other. Kang & Wheatley compared the pupil signal during dichotic presentation to the pupil signal during diotic presentation of the attended vs. ignored clip. Using dynamic time warping to determine the similarity between the pupillary signals of interest, they showed that in the dichotic condition, the pupil signal was more similar to the pupil signal recorded during diotic presentation of the attended clip than to that recorded during diotic presentation of the unattended clip (55). Such a finding implies that the pupil time series is a time-locked, continuous dependent measure that can reveal fine-grained information about an attended auditory stimulus. However, it remains to be determined whether it is possible for the pupil to become entrained to rhythmic auditory stimuli and whether such oscillations would reflect attentional processes or merely passive entrainment.

### Predicting dynamic auditory attention

Because the metric structure perceived by listeners is not readily derivable from the acoustic signal, a variety of algorithms have been developed to predict at what period listeners will perceive the beat. For example, most music software applications use algorithms to display tempo to users and a variety of contests exist in the music information retrieval community for developing the most accurate estimation of perceived tempo, as well as individual beats, e.g. the Music Information Retrieval Evaluation eXchange (MIREX) Audio Beat Tracking task (56). The beat period, however, it just one aspect of the musical meter. More sophisticated algorithms and models have been developed to predict all prominent metric periodicities in a stimulus, as well as the way in which attention might fluctuate as a function of the temporal structure of an audio stimulus, as predicted by Dynamic Attending Theory.

For instance, the Beyond-the-Beat (BTB) model (57) parses audio in a way analogous to the auditory nerve and uses a bank of 99 damped linear oscillators (reson filters) tuned to frequencies between 0.25 and 10 Hz to model the periodicities present in a stimulus. Several studies have shown that temporal regularities present in behavioral movement data (tapping and motion capture) collected from participants listening to musical stimuli correspond to the modeled BTB periodicity predictions for those same stimuli (57, 58, 59). Recent work (60) suggests that an additional model calculation of time-varying temporal salience can predict participants’ perceptual thresholds for detecting intensity changes at a variety of probed time points throughout the modeled stimuli, i.e. participants’ time-varying fluctuations in attention when listening to rhythmic patterns.

In the current study, we further tested the BTB model’s temporal salience predictions by asking whether output from the model could predict the pupillary response to rhythmic musical patterns. We hypothesized that the model could predict neurophysiological signals, such as the pupillary response, which we use as a proxy for attention. Specifically, we expected that the pupil would become entrained to the rhythmic musical patterns in a stimulus specific way.

We also expected to see phasic pupil dilation responses to intensity deviants. As in (60), we used an adaptive thresholding procedure to probe participants’ perceptual thresholds for detecting intensity increases (dB SPL) inserted at multiple time points throughout realistic, multi-part rhythmic stimuli. Each probed position within the stimulus had a corresponding value in terms of the model’s temporal salience predictions. We hypothesized that detection thresholds should be lower at moments of high model-predicted salience and vice versa. If perceptual thresholds differ for different moments in time, we assume this reflects fluctuations in attention, as predicted by DAT.

## Methods

### Participants

Eighteen people participated in the experiment (13 female; mean age: 26 years (min: 19; max: 52; median 23 years). Student participants from UC Davis received course credit for participation; other volunteer participants received no compensation. The experimental protocol was approved by the Institutional Review Board at UC Davis.

### Materials

**Figure 1.**
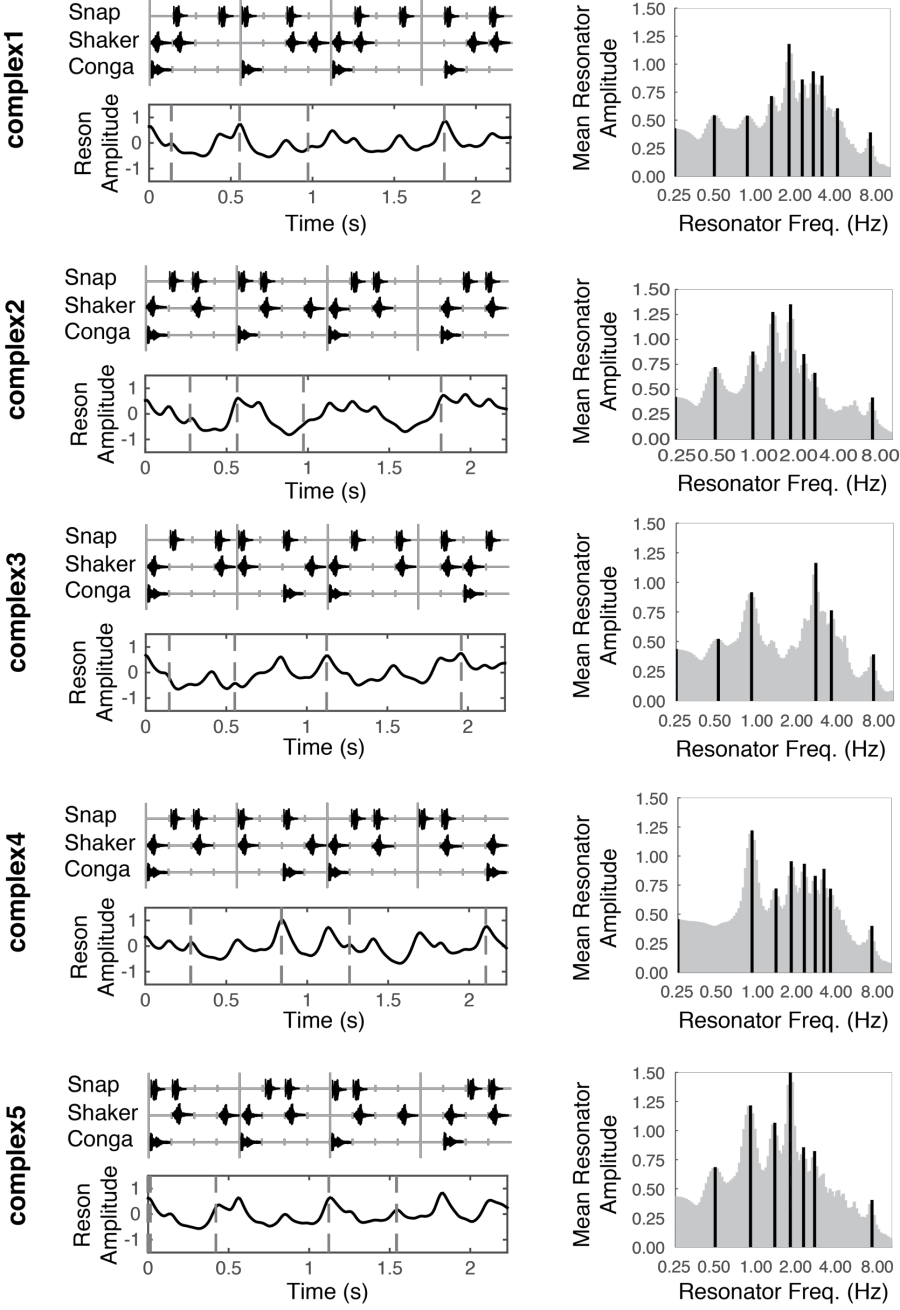
Stimulus patterns (left column, top panels), temporal salience predictions (left column, bottom panels), and mean periodicity profiles (right column) for each of the five stimuli used in this experiment. Vertical tick marks underlying the stimulus patterns (upper panels, left column) correspond to time in 140 ms intervals. Dotted vertical lines (bottom panels, left column), indicate moments in time that were probed with a deviant. These moments correspond to the musical event onset(s) directly above in the top panels. Dark vertical lines in the right column indicate peak periodicities in the mean periodicity profile.

The five rhythmic patterns used in this study (Fig. 1, left column, top panels) were initially created by Dr. Peter Keller via a custom audio sequencer in Max/MSP 4.5.7 (Cycling ‘74), for a previous experiment in our lab. Multi-timbre percussive patterns, each consisting of the snap, shaker, and conga samples from a Proteus 2000 sound module (E-mu Systems, Scotts Valley, CA) were designed to be played back in a continuous loop at 107 beats per minute, with a 4/4 meter in mind. However, we remain agnostic as the actual beat periodicity and metric periodicities listeners perceived in the stimuli, as we leave such predictions to the linear oscillator model. Each stimulus pattern lasted 2.2 s. We use the same stimulus names as in (60) for consistency. All stimuli can be accessed in the supplemental material of (60).

Please note that the intensity level changed dynamically throughout the experiment based on participants’ responses. The real-time, adaptive presentation of the stimuli is discussed further in the *Adaptive Thresholding Procedure* section below.

### Linear oscillator model predictions

All stimuli were processed through the Beyond-the-Beat model (57) to obtain mean periodicity profiles and temporal salience predictions. For full details about the architecture of the model and the periodicity surface calculations, see (57). For details about the temporal salience calculations, please see (60).

**Figure 2.**
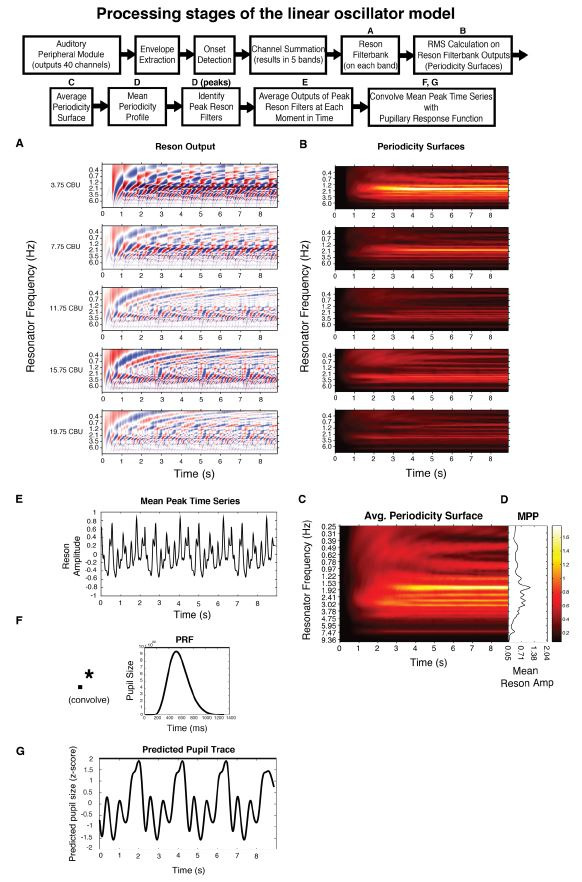
Boxes represent processing stages of the linear oscillator model; those labeled with a letter have a corresponding plot below. Please see *Linear oscillator model predictions* for full details. All plots are for stimulus *complex1.*

In short, the model uses the Institute for Psychoacoustics and Electronic Music toolbox (61) to transform the incoming audio in a manner analogous to the auditory nerve, separating the signal into 40 different frequency bands, with center frequencies ranging from 141 to 8877 Hz. Then, onset detection is performed in each band by taking the half-wave rectified first order difference of the root mean square (RMS) amplitude. Adjacent bands are averaged together to reduce redundancy and enhance computational efficiency. The signal from each of the remaining five bands is fed through a bank of 99 reson filters (linear oscillators) tuned to a range of frequencies up to 10 Hz. The oscillators driven most strongly by the incoming signal oscillate with the largest amplitude (Fig. 2A). A windowed RMS on the reson filter outputs results in five periodicity surfaces (one for each of the five bands), which show the energy output at each reson-filter periodicity (Fig. 2B). The periodicity surfaces are averaged together to produce an Average Periodicity Surface (Fig. 2C). The profile plotted to the right of each stimulus (Fig. 1, right column; Fig. 2D) is termed the Mean Periodicity Profile (MPP) and represents the energy at each periodicity frequency, averaged over time. Periodicities in the MPP that exceed 5% of the MPP’s amplitude range are considered peak periodicities and are plotted as dark black lines against the gray profile (Figure 1, right column).

After determining the peak periodicities for each stimulus, we return to the output in each of the five bands from the reson filters. We mask this output to only contain activity from the peak frequencies. Taking the point-wise mean resonator amplitude across the peak-frequency reson filters in all five bands yields the time series shown directly beneath each stimulus pattern in Figure 1 (also see Fig. 2E). We consider this output an estimate of salience over time.

In deciding the possible time points at which to probe perceptual thresholds, we tried to sample across the range of model-predicted salience values for each stimulus by choosing four temporal locations (dotted lines in lower panels of Figure 1). We treat the model predictions as a continuous variable.

To predict the temporal and spectral properties of the pupillary signal, we 1) extend the temporal salience prediction for multiple loop iterations 2) convolve the extended temporal salience prediction with a canonical pupillary response function (62) and 3) calculate the spectrum of this extended, convolved prediction. The pupillary response function (PRF) is plotted in Figure 2F. Its parameters have been empirically derived, first by (63) then refined by (62). The PRF is an Erlang gamma function, with the equation:

h = t^n^e ^(- nt/ t^_max_^)^

where *h* is the impulse response of the pupil, with latency *t_max_.* (63) derived *n* as 10.1 which represents the number of neural signaling steps between attentional pulse and pupillary response. They derived t_max_ as 930ms when participants responded to suprathreshold auditory tones with a button press. More recently, (62) estimated t_max_ to suprathreshold auditory tones in the absence of a button press to be 512ms. They show that this non-motor PRF is more accurate in correctly deconvolving precipitating attentional events and that it can be used even when there are occasional motor responses involved, e.g. responses to deviants, as long as they are balanced across conditions. Hence, in our case, we model the continuous pupillary response to our stimuli using the non-motor PRF and simply treat any motor responses to deviants as noise that is balanced across all of our conditions (stimuli).

Though previous studies have taken a deconvolution approach (deconvolving the recorded pupil data to get an estimate of the attentional pulses that elicited it), note that we here take a forward, convolutional approach. This allows us to generate predicted pupil data (Fig. 2G) which we compare to our recorded pupil data. With this approach, we avoid the issue of not being certain when exactly the attentional pulse occurred, i.e. with deconvolution it is unclear what the relationships are between the stimulus, attentional pulse, and the system’s delay (see discussion in (63) p. 24); also note that deconvolution approaches often require an additional temporal alignment technique such as an optimization algorithm, e.g. (64), and/or dynamic time-warping, e.g. (55). Here, we take the empirically derived delay, *t*, of the pupil to return to baseline from (62), Figure 1a as 1300ms.

### Alternative models

An important consideration is whether the complexity of the linear oscillator model is necessary to accurately predict behavioral and pupillary data for rhythmic stimuli. To address this question, two alternative models are considered in our analyses, each representing different, relevant aspects of the acoustic input sequence.

*Full resonator output:* Rather than masking the resonator output at the peak periodicities determined by the Mean Periodicity Profile to get an estimate of salience over time that is driven by the likely relevant metric frequencies, it is possible to just average the output from all reson filters over time. Such a prediction acts as a nice alternative to our filtered output and allows for a comparison of whether the prominent metric periodicities play a role in predicting attention over time.

*Amplitude Envelope:* The spectrum of the amplitude envelope of a sound signal has been shown to predict neural entrainment frequencies, e.g. (65) show cortical steady-state evoked potentials at peak frequencies in the envelope spectrum. Hence, as a comparison to the linear oscillator model predictions, we also used the amplitude envelope of our stimuli as a predictor. To extract the amplitude envelope of our stimuli, we repeated each stimulus for multiple loops and calculated the root mean square envelope using MATLAB’s *envelope* function with the ‘rms’ flag and a sliding window of 50ms. Proceeding with just the upper half of the envelope, we low-pass filtered the signal at 50 Hz using a 3^rd^ order Butterworth filter then down-sampled to 100 Hz to match the resolution of the oscillator model. To predict pupil data, we convolved the envelope with the PRF, as previously detailed for the linear oscillator model.

### Apparatus

Participants were tested individually in a dimly lit, sound-attenuating room, at a desk with a computer monitor, infrared eye-tracker, Logitech Z-4 speaker system, and a Dell keyboard connected to the computer via USB serial port. Participants were seated approximately 60 cm away from the monitor. Throughout the experiment, the screen was gray with a luminance of 17.7 cd/m^2^, a black fixation cross in the center, and a refresh rate of 60 Hz. The center to edge of the fixation cross subtended 2.8° of visual angle. Pupil diameter of the right eye was recorded with an Eyelink 1000 (SR Research) sampling at 500 Hz in remote mode, using Pupil-CR tracking and the ellipse pupil tracking model. Stimuli were presented at a comfortable listening level, individually selected by each participant, through speakers that were situated on the right and left sides of the computer monitor. During the experiment, auditory stimuli were adaptively presented through Max/MSP (Cycling ’74; code available at: https://github.com/janatalab/attmap.git ), which also recorded behavioral responses and sent event codes to the eye-tracking computer via a custom Python socket.

### Procedure

Participants were instructed to listen to the music, to maintain their gaze as comfortably as possible on the central fixation cross, and to press the “spacebar” key any time they heard an increase in volume (a deviant). They were informed that some increases might be larger or smaller than others and that they should respond to any such change. A 1 min practice run was delivered under the control of our experimental web interface, Ensemble (66), after which participants were asked if they had any questions.

During the experiment proper, a run was approximately 7 min long and consisted of approximately 190 repetitions of a stimulus pattern. There were no pauses between repetitions, thus a continuously looping musical scene was created. Please note that the exact number of loop repetitions any participant heard varied according to the adaptive procedure outlined below. Each participant heard each stimulus once, resulting in five total runs of approximately 7 min each, i.e. a roughly 35 min experiment.

Stimulus order was randomized throughout the experiment. Messages to take a break and continue when ready were presented after each run of each stimulus. Following the deviance detection task, participants completed questionnaires assessing musical experience, imagery abilities, genre preferences, etc. These questionnaire data were collected as part of larger ongoing projects in our lab and will not be reported in this study. In total, the experimental session lasted approximately 50 min; this includes the auditory task, self-determined breaks between runs (which were typically between 5-30 s), and the completion of surveys.

*Adaptive Thresholding Procedure:* Though participants experienced a continuous musical scene, we can think of each repetition of the stimulus loop as the fundamental organizing unit of the experiment that determined the occurrence of deviants. Specifically, after every standard (no-deviant) loop iteration, there was an 80% chance of a deviant, in one of the four probed temporal locations, without replacement, on the following loop iteration. After every deviant loop, there was a 100% chance of a no-deviant loop. The Zippy Estimation by Sequential Testing (ZEST) (67, 68) algorithm was used to dynamically change the decibel level of each deviant, depending on the participant’s prior responses and an estimated probability density function (p.d.f.) of their threshold.

The ZEST algorithm tracked thresholds for each of the four probed temporal locations separately during each stimulus run. A starting amplitude increase of 10 dB SPL was used as an initial difference limen. On subsequent trials, the p.d.f. for each probed location was calculated based on whether the participant detected the probe or not, within a 1000 ms window following probe onset. ZEST uses Bayes’ theorem to constantly reduce the variance of a posterior probability density function by reducing uncertainty in the participant’s threshold probability distribution, given the participant’s preceding performance. The mean of the resultant p.d.f. determines the magnitude of the following deviant at that location. The mean of the estimated probability density function on the last deviant trial is the participant’s estimated perceptual threshold.

Compared to a traditional staircase procedure, ZEST allows for relatively quick convergence on perceptual thresholds. Because the ZEST procedure aims to minimize variance, using reversals as a stopping rule, like in the case of a staircase procedure, does not make sense. Here, we used 20 observations as a stopping rule because (68) showed that 18 observations were sufficient in a similar auditory task and (60) demonstrated that, on average, 11 trials allowed for reliable estimation of perceptual threshold when using a dynamic stopping rule. For the current study, 20 was a conservative choice for estimating thresholds, which simultaneously enabled multiple observations over which to average pupillary data.

In summary, each participant was presented with a deviant at each of the four probed locations, in each stimulus, 20 times. The intensity change was always applied to the audio file for 200 ms in duration, i.e. participants heard an increase in volume of the on-going rhythmic pattern for 200 ms before the pattern returned to the initial listening volume. The dB SPL of each deviant was adjusted dynamically based on participants’ prior responses. The mean of the estimated probability density function on the last deviant trial (observation 20) was the participant’s estimated threshold. Examples of this adaptive stimulus presentation are accessible online as part of the Supplemental Material in (60):http://dx.doi.org/10.1037/xhp0000563.supp.

### Analysis

*Perceptual Thresholds:* Participants’ perceptual thresholds for detecting deviants at each of the probed temporal locations were computed via ZEST (67, 68); see the *Adaptive Thresholding Procedure* section above for further details.

*Reaction Time:* Reaction times were calculated for each trial for each participant, from deviant onset until button press. Trials containing reaction times that did not fall within three scaled median absolute deviations from the median were removed from subsequent analysis. This process resulted in the removal of 0.12% of the data.

*Pupil Preprocessing:* Blinks were identified in the pupil data using the Eyelink parser blink detection algorithm (69), which identifies blinks as periods of loss in pupil data surrounded by saccade detection, presumed to occur based on the sweep of the eyelid during the closing and opening of the eye. Saccades were also identified using Eyelink’s default algorithm.

Subsequently, all ocular data were preprocessed using custom scripts and third party toolboxes in MATLAB version 9.2 (70). Samples consisting of blinks or saccades were set to NaN, as was any sample that was 20 arbitrary units greater than the preceding sample. A sliding window of 25 samples (50ms) was used around all NaN events to remove edge artifacts. Missing pupil data were imputed by linear interpolation. Runs requiring 30% or more interpolation were discarded from future analysis, which equated to 9% of the data. The pupil time series for each participant, each run (~7 min), was high-pass filtered at .05 Hz, using a 3^rd^ order Butterworth filter, to remove any large-scale drift in the data. For each participant, each stimulus run, pupil data were normalized as follows: z-scoredPupilData = (rawData – mean(rawData)) / std(rawData). See Figure S1 in the Supplementary Materials accompanying this article for a visualization of these pupil pre-processing steps.

Collectively, the preprocessing procedures and some of the statistical analyses reported below relied on the Signal Processing Toolbox (v. 7.4), the Statistics and Machine Learning Toolbox (v. 11.1), the Bioinformatics Toolbox (v. 4.4), Ensemble (66), and the Janata Lab Music Toolbox (57, 71). All custom analysis code is available upon request.

*Pupil Dilation Response:* The pupil dilation response (PDR) was calculated for each probed deviant location in each stimulus by time-locking the pupil data to deviant onset. A baseline period was defined as 200 ms preceding deviant onset. The mean pupil size from the baseline period was subtracted from the trial pupil data (deviant onset through 3000 ms). The mean and max pupil size were calculated within this 3000 ms window. We chose 3000 ms because the pupil dilation response typically takes around 2500 ms to return to baseline following a motor response (62); therefore, 3000 ms seemed a safe window length. Additionally, the velocity of the change in pupil size from deviant onset to max dilation was calculated as the slope of a line fit from pupil size at deviant onset to max pupil size in the window, similar to Figure 1C in (72). The latency until pupil size maximum was defined as the duration (in ms) it took from deviant onset until the pupil reached its maximum size. Trials containing a baseline mean pupil size that was greater than three scaled median absolute deviations from the median were removed from subsequent analyses (0.2% of all trials).

*Time-Frequency Analyses:* To examine the spectro-temporal overlap between our varied model predictions and the observed pupillary signals, we calculated the spectrum of the average pupillary signal to 8-loop epochs of each stimulus, for each participant. We then averaged the power at each frequency across all participants, for each stimulus. We compared the continuous time series and the power spectral density for the recorded pupil signal for each stimulus to those predicted by the model predictions convolved with the pupillary response function. These two average analyses are included for illustrative purposes; note that the main analysis of interest is on the level of the single participant, single stimulus, as outlined below.

To compare the fine-grained similarity between the pupil size time series for any given stimulus to the linear oscillator model prediction for that stimulus, we computed the Cross Power Spectral Density (CPSD) between the pupil time series and itself, the model time series and itself, and the pupil time series and the model time series. The CPSD was calculated using Welch’s method (73), with a 4.4 s window and 75% overlap. For each participant, each stimulus, we computed 1) the CPSD between the pupil trace and the model prediction for that stimulus and 2) the CPSD between the pupil trace and the model prediction for all other stimuli, which served as the null distribution of coherence estimates.

The phase coherence between the pupil and the model for any given stimulus was defined as the squared absolute value of the pupil-model CPSD, divided by the power spectral density functions of the CPSD of the individual signals with themselves. We then calculated a single true and null coherence estimate for each participant, each stimulus, by finding the true vs. null coherence at each model-predicted peak frequency (see Table S2) under 3 Hz and averaging.

## Results

### Perceptual Thresholds

We tested each of the three alternative predictors of perceptual thresholds (peak filtered resonator output, full resonator output, and amplitude envelope) using mixed-effects models via the *nlme* package (74) in R (75). Threshold was the dependent variable and the given model’s predicted value at the time of each probe was the fixed effect. Random-effect intercepts were included for each participant. We calculated effect sizes of fixed effects using Cohen’s *f^2^*, a standardized measure of an independent variable’s effect size in the context of a multivariate model (76). We calculated *f*^2^ effect sizes following the guidelines of (77) for mixed-effects multiple regression models.

We assessed the relative goodness of model fit using Akaike’s information criterion (AIC; (78)). As widely recommended (e.g. (79)), we rescaled AIC values to represent the amount of information lost if choosing an alternative model, as opposed to the preferred model, with the equation:

∆_i_ = AIC_i_ - AIC_min_

where AIC_min_ is the model with the lowest AIC value of all models considered and AIC_i_ is the alternative model under consideration. Given this equation, AIC_min_, by definition, has a value of 0 and all other models are expressed in relation. Typically, models having a value ∆_i_ < 2 are considered to have strong support; models with 4 < ∆_i_ < 7 have less support, and models ∆_i_ > 10, no support. This transformation of the AIC value is important as it is free of scaling constants and sample size effects that influence the raw AIC score. In our case, because each of our models being compared has the same amount of complexity, Bayesian Information Criterion (80) differences are identical to those calculated for AIC ∆_i_ so we do not include them here.

As Table 1 indicates, our peak filtered model yielded the lowest AIC value, suggesting that it is strongly preferred over the amplitude envelope of the audio and entirely preferred over the full resonator model output as a predictor of perceptual threshold, given this common metrics of model fit. Furthermore, although full reson output and the amplitude envelope were both significant predictors of participant thresholds, the effect size was largest for the peak-filtered reson model compared to the alternatives. However, we note that no broadly accepted significance test exists for comparing non-nested mixed-effects models (i.e., each model contains a different fixed-effect term). As such, we caution that a strong claim of model-fit superiority would require further testing. Nevertheless, these results suggest that the peak-filtered resonator model better explains variance in participants’ thresholds than does the amplitude envelope of the auditory stimulus or the unfiltered reson output. We interpret this in favor of participants entraining their attention to endogenously generated metrical expectations which are represented by the peak periodicities from our model.

**Table 1.** Comparison of three alternative predictive models of perceptual threshold.

Threshold Predictor	ß	SE	*df*	*f^2^*	AIC	∆_i_
Peak-filtered reson	-3.30 **	.431	344	.17	2082.857	0
Full reson	-0.011**	.0023	344	.064	2125.737	42.8
Amplitude env	-133.14**	20.80	344	.12	2090.672	7.8

*Note.* Model estimates were obtained using linear mixed-effects models to regress fixed effects of stimulus model type on threshold; participant intercept was included as a random effect. AIC Akaike’s Information Criterion (a lower value indicates a more preferred model); Cohen’s *f^2^* for effect size. ** *p* < .001.

**Figure 3.**
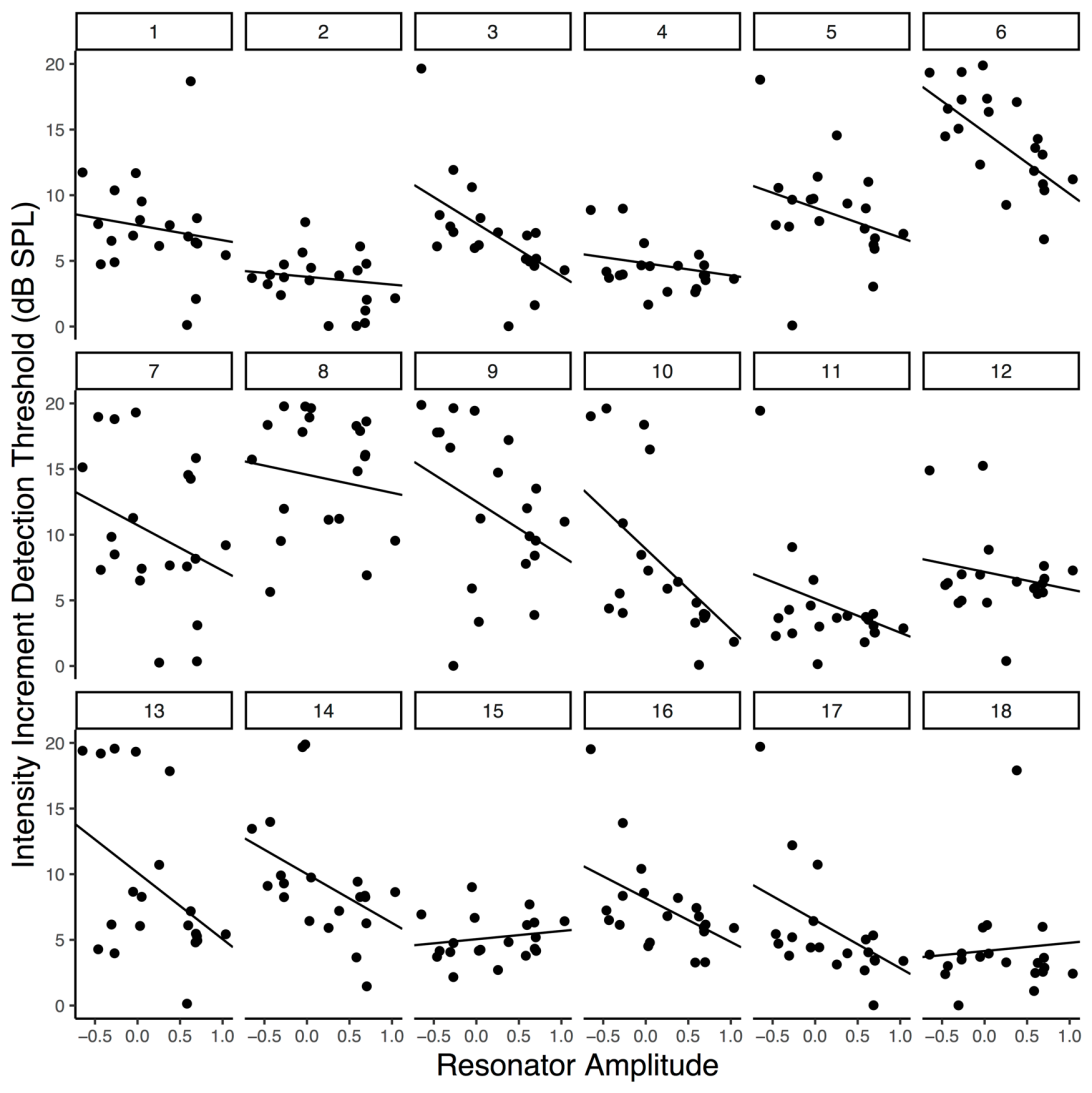
Increment detection thresholds as a function of averaged peak resonator level. Each panel is an individual participant’s data. Lines reflect participants’ slopes and intercepts as random effects in a mixed-effects model.

The negative relationship between peak resonator level and increment detection threshold is plotted in Figure 3 and visible within most participants’ data. Note that random slopes were included in the final model that generated Figure 3 so that participant level fit could be visualized and because the there is growing consensus that the random effects structure of linear mixed effects models should be kept maximal (81); however, we wish to note that adding the random slope did not significantly improve the fit of the model, which is why it was not included during our model comparisons. Overall, the final peak reson filtered model had a conditional R^2^ of .087 – reflecting an approximation of the variance explained by the fixed effect of peak reson output – and a marginal R^2^ of .475 – reflecting an approximation of the variance explained by the overall model (fixed effect of peak reson output plus random intercepts and slopes for participants). R^2^ estimates were calculated using the *MuMIn* package in R (82, 83, 84).

In the remainder of the paper, we refrain from using the alternative full reson model, as it could be confidently rejected and was weakest of the three models. We do continue to compare the peak filtered model with the amplitude envelope; however, we wish to note that a strong correlation exists between the amplitude envelope predictions and the peak-filtered predictions at each probed position (r(20) = .90, *p* < .001).

### Pupil Dilation Response (PDR)

**Figure 4.**
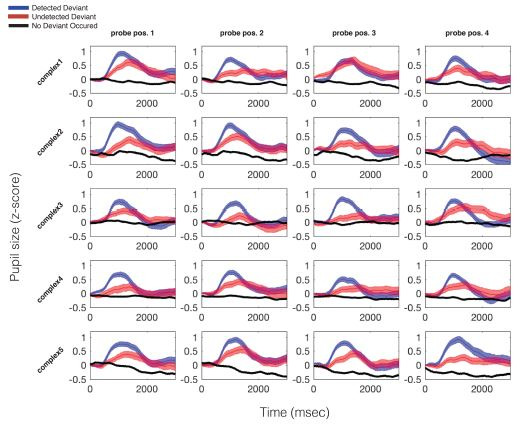
Average pupillary responses, across all participants, to all probed locations in all stimuli. The blue trace indicates the average pupillary response to trials during which a deviant occurred and was detected; the red trace represents trials during which a deviant occurred but was not detected; the black trace indicates the same moments in time on trials in which no deviant occurred. All data are time locked to deviant onset. For no-deviant trials, this refers to the same point in time that the deviant could have occurred (but did not). Width of each trace is the standard error of the mean.

The average pupil dilation response for each trial type, for each probed position, in each stimulus, is plotted in Figure 4. Possible trial types are 1) trials in which a deviant occurred and was detected by participants (blue), 2) trials in which a deviant occurred and was not detected by participants (red), and 3) trials in which no deviant occurred (black). In all cases the data are plotted with respect to the probed time point. Please recall that the only difference between deviant and no-deviant trials is that the auditory event at the probed moment in time is increased in dB SPL, relative to the baseline volume of the standard pattern. On average, per participant, per probe position, 12 trials were used in calculating the “hit” average, and 8 trials were used in calculating the “miss” average. For a full report of the average number and standard deviation of trials used in calculating each grand average trace plotted in Figure 4, see Table S1.

As can be seen in Figure 4, the PDR to a deviant is consistent and stereotyped across all probed time points There was a significant difference between mean pupil size on hit vs. missed trials, t(15) = 2.14, *p* = .049, and missed vs. no deviant trials, t(15) = 4.60, *p* < .001. Additional features of the PDR also varied as a function of whether the deviant was detected or missed. There was a significant difference between max pupil size on hit vs. missed trials, t(15) = 4.48, *p* < .001, and missed vs. no-deviant trials, t(15) = 28.45, *p* < .001, as well as a marginally significant difference between pupil latency until maximum on hit vs. missed trials t(15) = -2.11, *p* = .052. There was no significant difference between pupil velocity to maximum on hit vs. miss trials.

Before constructing trial-level predictive models based on specific features of the PDR, we first assessed correlations between all predictor and outcome variables. All Pearson correlation coefficients for both hit and miss trials, across all participants, are reported in Table 2. We found that baseline pupil size is negatively correlated with mean and max evoked pupil size, as well as latency to max pupil size and pupil velocity. Mean evoked pupil size – the standard metric in most cognitive studies – was strongly positively correlated with max evoked pupil size, latency to max pupil size, and pupil velocity. It is also worth noting that decibel contrast (relative to baseline volume) was negatively correlated with reson output, reflecting our perceptual threshold results, i.e. moments of low salience required higher dB contrast to be detected. There was no correlation between dB contrast and pupil dilation, adding to the literature of mixed findings on this topic. Reaction time was weakly correlated with baseline pupil size, dB contrast, and max evoked pupil size, though not mean evoked pupil size, despite the strong correlation of these two pupil variables with each other. As the coefficients indicate, these reaction time effects are very weak at best, possibly due to our use of a standard computer keyboard which may have introduced jitter in the recording of responses.

Because of the strong correlations between possible pupil metrics of interest, in subsequent analyses we used maximum evoked pupil size in our statistical models, so as not to construct models with collinear predictors. While it may be argued that baseline pupil size is a more intuitive metric to use, as it could indicate causality, we wish to point out that there were no significant differences in baseline pupil size between hit vs. missed trials t(15) = -0.75, *p* = .463, while there was a significant difference in max evoked pupil size between hit and missed trials, as previously indicated. Hence, though baseline pupil size is strongly correlated with mean and max evoked pupil size, it is not our predictor of interest in context of this analysis.

To test if we could predict whether a deviant was detected or not based on the evoked pupillary response, on a per-trial basis, we fit a generalized linear mixed-effects logistic regression model. The generalized linear mixed-effects model (GLMM) included max evoked pupil size and peak reson output as predictors, participant as a random intercept, and a binary *hit* (detection of the increment) or *miss* (non-detection of the increment) as the dependent variable. The GLMM was fit via maximum likelihood using the Laplace approximation method and implemented using the *glmer* function from the lme4 package (85) in R. Odds ratios, z statistics, confidence intervals, and p-values are reported for all fixed effects (Table 3).

As a comparison, we ran the same model but swapped peak reson prediction for amplitude envelope prediction. We used the *pROC* package (86) to calculate the Receiver Operating Characteristic (ROC) curves. ROC curves compare the true positive prediction rate against the false positive rate. We compared the area under the ROC curves (AUC) of the peak reson model vs. the amplitude envelope model using DeLong’s test for two correlated ROC curves (87). The AUC is a common metric for evaluating both the goodness of fit of a model, as well as the performance of two different models. In this case, it reflects the probability that a randomly selected ‘hit’ trial is correctly identified as a ‘hit’ rather than ‘miss’ (88). With a range of .5 (chance) to 1 (perfect prediction), higher AUC values indicate better model performance. The peak reson model had an AUC of .608, while the amplitude envelope model had an AUC of .606, thus there was no significant difference between the models (Z = 1.594, *p* = .111). Both performed significantly above chance, whether chance was defined as the standard .5 or more conservatively via shuffling of the pupil and model data (peak reson vs. shuffled chance: Z = 3.79, *p* < .001; amp env vs. shuffled chance: Z = 3.53, *p* < .001) .

Given the similar performance of peak reson and amplitude envelope models, and the fact that the pupil dilation response to deviants at different moments of predicted salience is remarkably stereotyped, it is not possible to be sure whether the pupil dilation response reflects endogenous meter perception or merely a bottom-up response to the stimuli. An experiment with a wider range of stimuli, incorporating non-stationary rhythms, might be well suited to answer this question, as such stimuli would likely result in a greater difference between the amplitude envelope and the peak reson filter predictions. Nonetheless, the finding of a PDR on trials in which participants failed to report detecting a deviant has implications for future studies and is discussed in more detail below.

**Table 2.** Pearson Correlation Coefficients for all predictor and outcome variables on ‘hit’ and ‘miss’ trials.

Variables	1 Hit (Miss)	2	3	4	5	6	7
1. Mean baseline pup	-						
2. Decibel contrast	.004 (0)	-					
3. Resonator level	.012 (0.004)	-.185**(-.338)**	-				
4. Mean evoked pup	-.811** (-.787)**	-.001 (.004)	-.022 (.01)	-			
5. Max evoked pup	-.728** (-.742)**	-.009 (.009)	-.015 (-.01)	.868** (.887**)	-		
6. Max latency	-.443** (-.492)**	.023 (-.019)	.015 (-.026)	.443** (.466**)	.357** (.417**)	-	
7. Pup velocity	-.144** (-.142)**	-.055* (.013)	.008 (.023)	.245** (.230**)	.219** (.196**)	.163** (.168**)	-
8. Reaction time	.045* (-)	.077** (-)	.006 (-)	-.01 (-)	-.039* (-)	.011 (-)	-.003 (-)

*Note.* ‘Miss’ trial correlation coefficients are in parenthesis; there is no reaction time for a ‘miss’ trial. Pup refers to pupil size. Decibel contrast is the change in dB, relative to baseline volume, of a deviant on any given trial. For the ‘hit’ data, df = 3108, for the ‘miss’ data, df = 2167, *p < .05, **p < .001

**Table 3.** Generalized linear mixed-effects logistic regression model with dependent variable hit (1) or miss (0).

			95% CI for OR	
	OR	*Z* stat	Lower	Upper
Max pupil size	1.28 **	8.35	1.21	1.16
Resonator output	1.26 **	3.86	1.12	1.41

*Note*. 5279 observations. OR = Odds Ratio*.* CI = Confidence Interval. **p < .001

### Continuous pupil signal

While the pupil dilation response to deviants is of interest with regards to indexing change detection, we also wished to examine the continuous pupillary response to our rhythmic stimuli. Specifically, we wanted to assess whether the pupil entrained to the rhythmic patterns and, if so, whether such dynamic changes in pupil size were unique for each stimulus.

**Figure 5.**
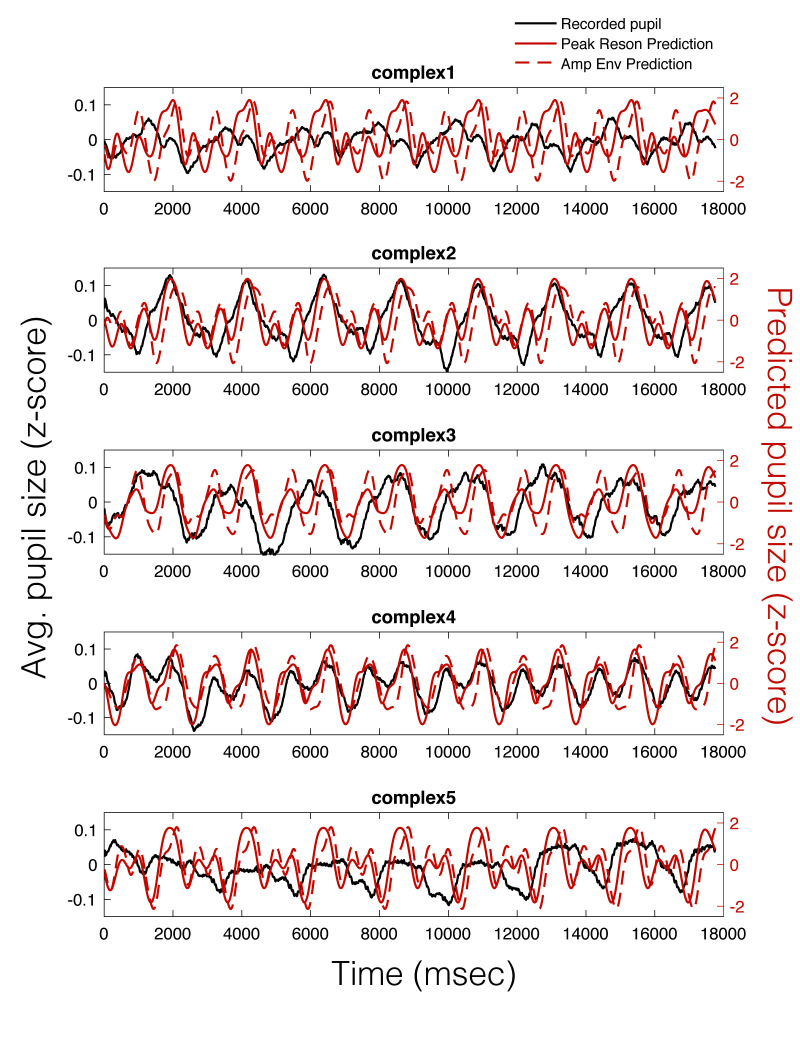
Average pupillary responses, across all participants, to 8-loop epochs of each stimulus (black), compared to the peak reson filter model-predicted pupillary responses (solid red) and the amplitude envelope-predicted pupillary responses (dotted red).

**Figure 6.**
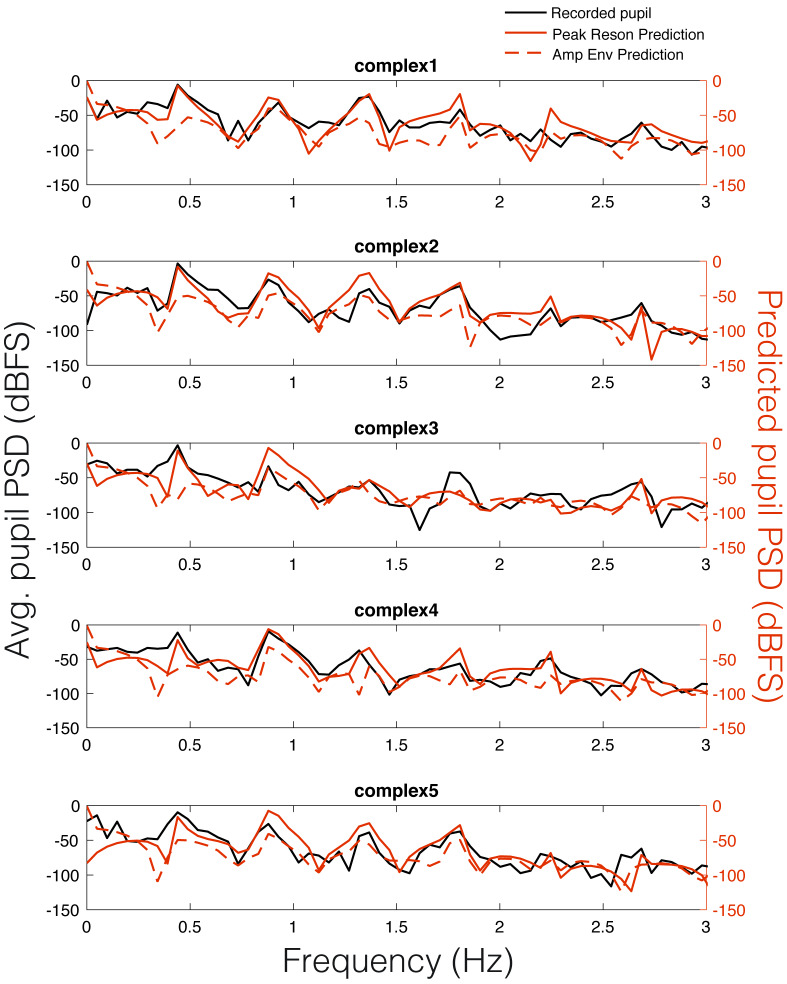
Average pupillary power spectral density (PSD) across participants (black) vs. peak reson filter model-predicted PSD for each stimulus (solid red) and amplitude envelope-predicted PSD (dashed red).

As can be seen in Figure 5, there appears to be a stimulus-specific correspondence between the model predicted pupil time series (red) and the observed continuous pupillary signal (black). Note the remarkably similar predictions of the peak-filtered oscillator model (solid red) and the amplitude envelope model (dashed red). This correspondence between the two predictions and the recorded pupil data was also observable in the Power Spectral Density (PSD) estimates for each stimulus (Figure 6). Similar to studies of pupil oscillations in the visual domain (39), we do not see much power in the pupil spectrum beyond about 3 Hz; therefore, we plot frequencies in the range of 0 to 3 Hz (Figure 6). In Figure 6, it is clear that the spectra of the pupil signal and that of the two model predictions overlap. However, this analysis is not sufficient to infer that the pupil is tracking our model output in a stimulus-specific way, though it does indicate pupillary entrainment to the prominent periodicities in the stimuli.

To examine the relationship between each participant’s pupil time series and the model prediction for each stimulus, we examined their phase coherence. Because the peak reson and amplitude envelope models are very similar in temporal and spectral realms, we chose to compute phase coherence for only the peak reson model. An additional reason for doing this was because the peak reson model outputs predicted salient metric frequencies at which we can calculate coherence on a theoretical basis, whereas, with the amplitude envelope model, one would have to devise a comparable method to pick peaks in the envelope spectrum and ensure that they are of a similar number, spacing, and theoretical relevance.

**Figure 7.**
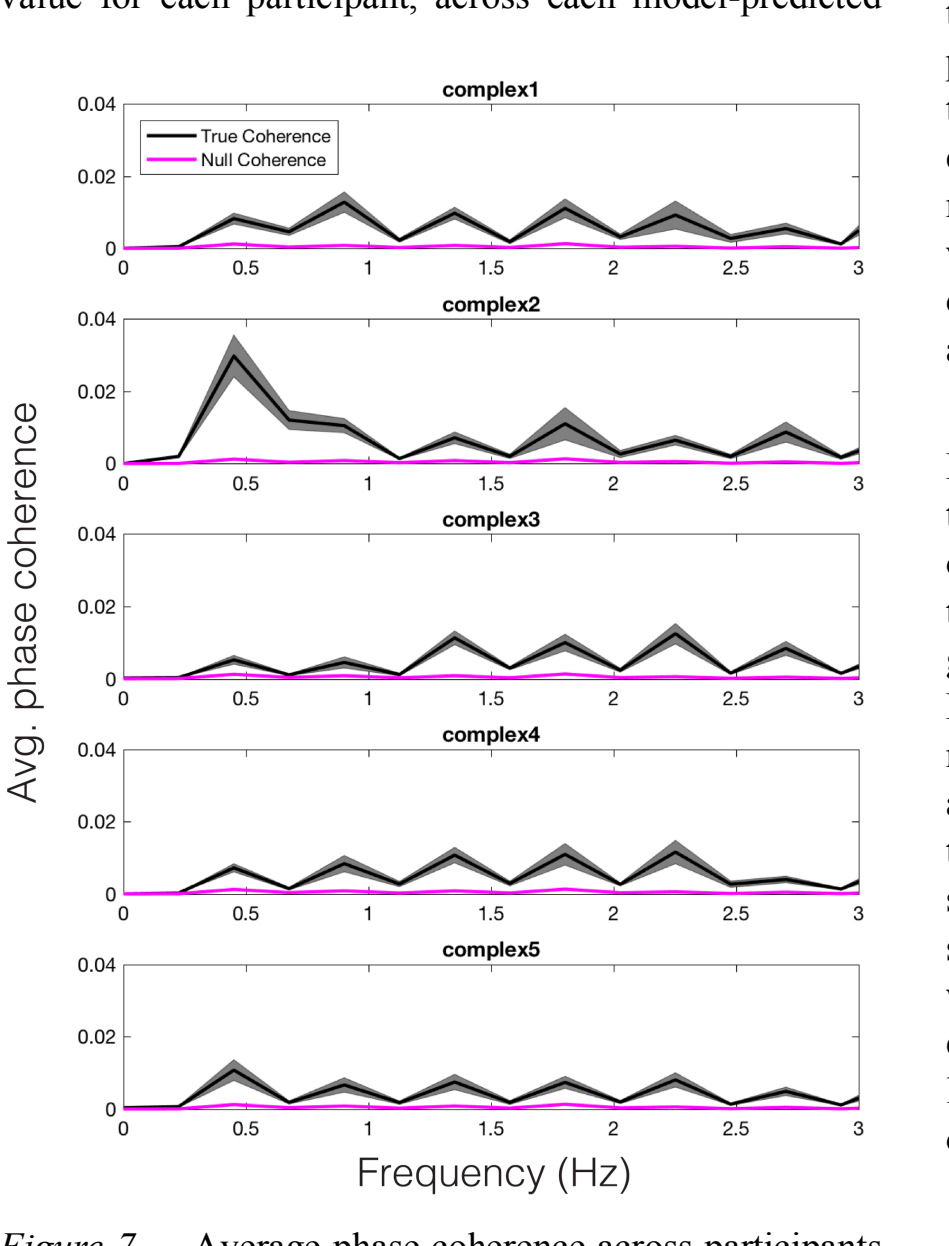
Average phase coherence across participants between the pupil and peak reson filter model, for each stimulus (black). Average null coherence is plotted in magenta. The width of each trace represents standard error of the mean.

Shuffling the stimulus labels allowed us to compute null coherence estimates (see *Time Frequency Analyses* for more details)*.* The average true vs. null coherence value for each participant, across each model-predicted peak frequency, was subjected to a paired samples t-test, revealing a significant difference in the true vs. null distributions for the peak reson-filtered model, t(14) = 16.56, *p* < .001. Thus, we can conclude that the changing dynamics of pupil size were entrained to the model predictions for each stimulus in a unique way. For illustration, we have plotted the average coherence, across participants, for each stimulus, in Figure 7 (black). The null coherence estimate is plotted in magenta. We wish to note that similar results would have likely been obtained via calculating the coherence between the amplitude envelope model and the pupil signal.

## Discussion

The current experiment used a linear oscillator model to predict both perceptual detection thresholds and the pupil signal during a continuous auditory psychophysical task. During the task, participants listened to repeating percussion loops and detected momentary intensity increments. We hypothesized that the linear oscillator model would predict perceptual thresholds for detecting intensity deviants that were adaptively embedded into our stimuli, as well as the continuous pupillary response to the stimuli.

The linear oscillator model reflects the predictions of Dynamic Attending Theory (DAT), which posits that attention can become entrained by an external (quasi)-periodic stimulus. The model is driven largely by onsets detected in the acoustic envelope of the input signal, which get fed through a bank of linear oscillators (reson filters). From there it is possible to calculate which oscillators are most active, mask the output at those peak frequencies, and average over time. Throughout the paper, we considered this peak-filtered signal the ideal prediction of temporal salience, as in (60); however, for comparison, we also tested how well output from all resonators would predict our data, as well as how well the amplitude envelope alone (without any processing through the oscillator model) would do in predicting both perceptual thresholds and the pupillary signal.

The peak-filtered model was best at predicting perceptual thresholds, providing an important replication and extension of our previous study (60). In the present study we used only complex stimuli and intensity increments but our previous study showed the same predictive effects of the peak-filtered model for both intensity increments and decrements, as well as simple and complex stimuli (60). We assume that such results imply that the peaks extracted by our model are attentionally relevant periodicities that guide listeners’ attention throughout complex auditory scenes. The fact that perceptual thresholds were higher at moments of low predicted salience, i.e. a deviant needed to be louder at that moment in time for participants to hear it and vice versa, indicates that attention is not evenly distributed throughout time, in line with the predictions of DAT. However, we note that the linear oscillator model’s temporal salience prediction was strongly correlated with the magnitude of the amplitude envelope of the signal.

Indeed, when it comes to the pupil signal, both the peak-filtered model and the amplitude envelope performed almost identically. This similarity is likely a result of a variety of factors: 1) the rhythms used in the current study are stationary (unchanging over time) and, though there are some moments lacking acoustic energy, overall, the prominent periodicities are present in the acoustic signal. Hence, the Fourier Transform of the amplitude envelope yields roughly identical peak periodicities to that of the model. 2) Convolving both signals with the pupillary response function smears out most subtle differences between the two signals, making them even more similar.

Regardless of the ambiguity regarding which model may be a better predictor, the pupillary results reported in this paper are exciting nonetheless. First and foremost, we show that the pupil can entrain to a rhythmic auditory stimulus. To our knowledge, we are the first to report such a finding, though others have reported pupillary entrainment in the visual domain (39, 40). The continuous pupillary signal and the pupil spectrum to each stimulus were both well predicted by the linear oscillator model and the amplitude envelope of the audio signal. That pupil dilation/constriction dynamics, controlled by the smooth dilator and sphincter muscles of the iris, respectively, entrain to auditory stimuli is in line with a large literature on music-evoked entrainment. Though the pupil has never been mentioned in this literature, other areas of the autonomic nervous system have been shown to entrain to music (41). It remains to be tested how pupillary oscillations might relate to cortical neural oscillations, as highlighted in the introduction of this paper. Are pupillary delta oscillations phase-locked to cortical delta? Do cortical steady-state evoked potentials overlap with those of the pupil? Pupillometry is a more mobile and cost-effective method than EEG, as such, characterizing the relationship between pupillary and cortical responses to music will hopefully allow future studies to use pupillometry in situations that otherwise might have required EEG.

Furthermore, we have shown not only that the pupil entrains to the prominent periodicities present in our stimuli, but also that the oscillatory pupillary response to each stimulus is unique. These results extend those of (55) and speak to the effectiveness of using pupillometry in the context of music cognition studies. Unlike (55), we did not use deconvolution or dynamic time-warping to assess the fit of our pupil data with our stimuli, rather, we took a forward approach to modeling our stimuli, convolving stimulus-specific predictions with a pupillary response function, effectively removing the need for algorithms like dynamic time-warping or fitting optimizations. We hope that this approach will prove beneficial for others, especially given the simplicity in calculating the amplitude envelope of a signal and convolving it with the pupillary response function. With regards to our linear oscillator model, future work will use a wider and more temporally dynamic variety of stimuli to assess the power of our linear oscillator model vs. the amplitude envelope in predicting the time-varying pupil signal across a diverse range of musical cases. Hopefully such a study will shed more light on the issue of whether pupillary oscillations are an evoked response or a reflection of attention and in what contexts one might need to use a more complex model, if any.

Even in the case of oscillations being driven more so by the stimuli than by endogenous attention, we still feel that such oscillations nevertheless shape subsequent input and reflect, in some way, the likely attended features of the auditory, and perhaps visual, input. Because pupil dilation blurs the retinal image and widens the visual receptive field, while pupil constriction sharpens the retinal image, narrowing the visual receptive field (40), oscillations in pupil size, which are driven by auditory stimuli may also have ramifications for visual attention (89) and audiovisual integration. For example, visual attention should be more spatially spread (greater sensitivity) at moments of greater auditory temporal salience (larger pupil dilation). It is possible, however, that such small changes in pupil size elicited by music are negligible with respect to visual sensitivity and/or acuity (see (90) for a discussion). In short, such interactions remain to be empirically tested.

Another important finding of the current study is the pupil dilation response (PDR) to deviants. Of particular interest is the result that the pupil responds to deviants even when participants do not report hearing them, providing further evidence that pupillary responses are indicators of preconscious processing (91). However, the current results raise an additional important question of whether the PDR might be more akin to the mismatch negativity (MMN) than the P3, which requires conscious attention to be elicited (92). Others have shown a PDR to deviants in the absence of attention (51, 54) and here we show a PDR to deviants that did not reach participants’ decision thresholds, or possibly conscious awareness, despite their focused attention. Hence, though there is evidence connecting the P3a to the PDR and the LC-NE system (43, 93), an important avenue of future research will be to disentangle the relationship between the PDR, P3a, and MMN, which can occur without the involvement of conscious attention (94) or perception (95). While all three of these measures can be used as indices of deviance detection, the P3a has been proposed to reflect comparison of sensory input with a previously formed mental expectation that is distributed across sensory and motor regions (96, 97), whereas the MMN and PDR have been interpreted as more sensory-driven, pre-attentive comparison processes.

Though we are enthusiastic about the PDR results, a few considerations remain. Since the present experiment only utilized intensity increments as deviants, it could be argued that the pupil dilation response observed on trials during which a deviant was presented below perceptual threshold does not reflect subthreshold processes but rather a linear response to stimulus amplitude. To this argument, we point to the fact that there was no correlation between the mean or max evoked pupil size on any given trial and the contrast in dB SPL on that trial. However, to further address this possible alternative interpretation, we conducted an experiment involving both intensity increments and decrements. Those data show the same PDR patterns for both increments and decrements, hit vs. missed trials, as reported in the current study (98). In addition, previous studies of rhythmic violations showed a standard PDR to the omission of an event (e.g. (54)), suggesting that the PDR is not specific to intensity increment deviance.

An additional critique might be that the difference between the pupil size on hit vs. missed trials is because hit trials require a button press while miss trials do not. Though it may be the case that the additional motor response required to report deviance detection results in a larger pupil size, this is unlikely to fully account for the difference in results (52, 53). Critically, even if a button press results in a greater pupil dilation, this does not change the fact that a PDR is observed on trials in which a deviant was presented but not reported as heard (uncontaminated by a button press).

In summary, our study contributes to a growing literature emphasizing the benefits of using eye-tracking in musical contexts (for further examples, please see the other articles in this Special Issue on Music & Eye-Tracking). We have shown that the pupil of the eye can reliably index deviance detection, as well as rhythmic entrainment. Considered in conjunction with previous studies from our lab, the linear oscillator model utilized in this paper is a valuable predictor on multiple scales – tapping and large body movements (57, 58, 59), perceptual thresholds (60), and pupil dynamics (current study). In general, the model can explain aspects of motor entrainment within a range of .25 – 10 Hz – the typical range of human motor movements. Future work should further compare the strengths and limitations of models of rhythmic attending (e.g. (27, 99)), the added benefits of such models over simpler predictors such as the amplitude envelope, and the musical contexts in which one model is more effective than another.

## Ethics and Conflict of Interest

The authors declare that the contents of the article are in agreement with the ethics described in http://biblio.unibe.ch/portale/elibrary/BOP/jemr/ethics.html and that there is no conflict of interest regarding the publication of this paper.

## Acknowledgements

This research was supported in part by LF’s Neuroscience Graduate Group fellowship, ARCS Foundation scholarship, and Ling-Lie Chau Student Award for Brain Research, as well as a Templeton Advanced Research Program grant from the Metanexus Institute to PJ.

We wish to thank Dr. Jeff Rector for providing the communication layer between the MAX/MSP software and the eye-tracking computer, and Dr. Peter Keller for constructing the rhythmic patterns used in this study.

## References

[b78] Akaike, H. (1974). A new look at the statistical model identification. IEEE Transactions on Automatic Control, 19(6), 716–723. 10.1109/TAC.1974.1100705 0018-9286

[b95] Allen, J., Kraus, N., & Bradlow, A. (2000). Neural representation of consciously imperceptible speech sound differences. Perception & Psychophysics, 62(7), 1383–1393. 10.3758/BF03212140 0031-5117 11143450

[b35] Aston-Jones, G., Rajkowski, J., Kubiak, P., & Alexinsky, T. (1994). Locus coeruleus neurons in monkey are selectively activated by attended cues in a vigilance task. The Journal of Neuroscience : The Official Journal of the Society for Neuroscience, 14(7), 4467–4480. 10.1523/JNEUROSCI.14-07-04467.1994 0270-6474 8027789 PMC6577022

[b81] Barr DJ, Levy R, Scheepers C, Tily HJ Random effects structure for confirmatory hypothesis testing: Keep it maximal. J Mem Lang. Elsevier Inc.; 2013;68(3):255–78. 10.1016/j.jml.2012.11.001 PMC3881361 24403724

[b85] Bates, D., Maechler, M., Bolker, B., & Walker, S. (2015). Fitting linear mixed-effects models using lme4. Journal of Statistical Software, 67(1), 1–48. 10.18637/jss.v067.i01 1548-7660

[b49] Beatty, J. (1982). Phasic not tonic pupillary responses vary with auditory vigilance performance. Pscyhophysiology., 19(2), 167–172. 10.1111/j.1469-8986.1982.tb02540.x 0048-5772 7071295

[b34] Berger, C. C., & Ehrsson, H. H. (2013). Mental imagery changes multisensory perception. Current Biology, 23(14), 1367–1372. 10.1016/j.cub.2013.06.012 0960-9822 23810539

[b2] Bergeson, T. R., & Trehub, S. E. (2006). Infants Perception of Rhythmic Patterns. Music Percept An Interdiscip J., 23(4), 345–360. 10.1525/mp.2006.23.4.345

[b36] Berridge, C. W., & Waterhouse, B. D. (2003). The locus coeruleus-noradrenergic system: Modulation of behavioral state and state-dependent cognitive processes. Brain Research. Brain Research Reviews, 42(1), 33–84. 10.1016/S0165-0173(03)00143-7 0165-0173 12668290

[b7] Bolger, D., Coull, J. T., & Schön, D. (2014). Metrical rhythm implicitly orients attention in time as indexed by improved target detection and left inferior parietal activation. Journal of Cognitive Neuroscience, 26(3), 593–605. 10.1162/jocn_a_00511 0898-929X 24168222

[b6] Bolger, D., Trost, W., & Schön, D. (2013). Rhythm implicitly affects temporal orienting of attention across modalities. Acta Psychologica, 142(2), 238–244. 10.1016/j.actpsy.2012.11.012 0001-6918 23357092

[b79] Burnham, K. P., & Anderson, D. R. (2004). Multimodel inference: Understanding AIC and BIC in model selection. Sociological Methods & Research, 33(2), 261–304. 10.1177/0049124104268644 0049-1241

[b19] Buzsaki G. Neuronal Oscillations in Cortical Networks. Science (80-) . 2004;304:1926–1929. 10.1126/science.1099745 15218136

[b5] Cason, N., & Schön, D. (2012). Rhythmic priming enhances the phonological processing of speech. Neuropsychologia, 50(11), 2652–2658. 10.1016/j.neuropsychologia.2012.07.018 0028-3932 22828660

[b76] Cohen, J. (1988). Statistical power analysis for the behavioral sciences. New York, NY: Routledge.

[b54] Damsma, A., & van Rijn, H. (2017). Pupillary response indexes the metrical hierarchy of unattended rhythmic violations. Brain and Cognition, 111, 95–103. 10.1016/j.bandc.2016.10.004 0278-2626 27816784

[b40] Daniels, L. B., Nichols, D. F., Seifert, M. S., & Hock, H. S. (2012). Changes in pupil diameter entrained by cortically initiated changes in attention. Visual Neuroscience, 29(2), 131–142. 10.1017/S0952523812000077 0952-5238 22391296

[b56] Davies MEP, Degara N, Plumbley MD Evaluation Methods for Musical Audio Beat Tracking Algorithms. Tech Rep C4DM-TR-09-06 8 Oct 2009. 2009;(10):17.

[b87] DeLong, E. R., DeLong, D. M., & Clarke-Pearson, D. L. (1988). Comparing the areas under two or more correlated receiver operating characteristic curves: A nonparametric approach. Biometrics, 44(3), 837–845. 10.2307/2531595 0006-341X 3203132

[b52] Einhäuser, W., Stout, J., Koch, C., & Carter, O. (2008). Pupil dilation reflects perceptual selection and predicts subsequent stability in perceptual rivalry. Proceedings of the National Academy of Sciences of the United States of America, 105(5), 1704–1709. 10.1073/pnas.0707727105 0027-8424 18250340 PMC2234208

[b8] Escoffier, N., Sheng, D. Y., & Schirmer, A. (2010). Unattended musical beats enhance visual processing. Acta Psychologica, 135(1), 12–16. 10.1016/j.actpsy.2010.04.005 0001-6918 20451167

[b98] Fink L, Hurley B, Geng J, Janata P Predicting attention to auditory rhythms using a linear oscillator model and pupillometry. In: Proceedings of the Conference on Music & Eye-Tracking. Frankfurt, Germany; 2017.

[b99] Forth, J., Agres, K., Purver, M., & Wiggins, G. A. (2016). Entraining IDyOT: Timing in the Information Dynamics of Thinking. Frontiers in Psychology, 7, 1575. 10.3389/fpsyg.2016.01575 1664-1078 27803682 PMC5067415

[b46] Gingras, B., Marin, M. M., Puig-Waldmüller, E., & Fitch, W. T. (2015). The Eye is Listening: Music-Induced Arousal and Individual Differences Predict Pupillary Responses. Frontiers in Human Neuroscience, 9, 619. 10.3389/fnhum.2015.00619 1662-5161 26617511 PMC4639616

[b9] Grahn, J. A. (2012). See what I hear? Beat perception in auditory and visual rhythms. Experimental Brain Research, 220(1), 51–61. 10.1007/s00221-012-3114-8 0014-4819 22623092

[b10] Grahn, J. A., Henry, M. J., & McAuley, J. D. (2011). FMRI investigation of cross-modal interactions in beat perception: Audition primes vision, but not vice versa. NeuroImage, 54(2), 1231–1243. 10.1016/j.neuroimage.2010.09.033 1053-8119 20858544 PMC3002396

[b25] Grahn, J. A., & Rowe, J. B. (2013). Finding and feeling the musical beat: Striatal dissociations between detection and prediction of regularity. Cerebral Cortex (New York, N.Y.), 23(4), 913–921. 10.1093/cercor/bhs083 1047-3211 22499797 PMC3593578

[b88] Green, D., & Swets, J. (1996). Signal detection theory and psychophysics. New York: John Wiley and Sons.

[b21] Henry, M. J., & Herrmann, B. (2014). Low-Frequency Neural Oscillations Support Dynamic Attending in Temporal Context. Timing & Time Perception (Leiden, Netherlands), 2(1), 62–86. 10.1163/22134468-00002011 2213-445X

[b63] Hoeks, B., & Levelt, W. J. M. (1993). Pupillary dilation as a measure of attention: A quantitative system analysis. Behavior Research Methods, Instruments, & Computers, 25(1), 16–26. 10.3758/BF03204445 0743-3808

[b50] Hong, L., Walz, J. M., & Sajda, P. (2014). Your eyes give you away: Prestimulus changes in pupil diameter correlate with poststimulus task-related EEG dynamics. PLoS One, 9(3), e91321. 10.1371/journal.pone.0091321 1932-6203 24618591 PMC3950210

[b11] Hove, M. J., Fairhurst, M. T., Kotz, S. A., & Keller, P. E. (2013). Synchronizing with auditory and visual rhythms: An fMRI assessment of modality differences and modality appropriateness. NeuroImage, 67, 313–321. 10.1016/j.neuroimage.2012.11.032 1053-8119 23207574

[b60] Hurley BK, Fink LK, Janata P Mapping the Dynamic Allocation of Temporal Attention in Musical Patterns Musical Patterns. J Exp Psychol Hum Percept Perform. 2018;Advance On. 10.1037/xhp0000563 30091636

[b58] Hurley, B. K., Martens, P. A., & Janata, P. (2014). Spontaneous sensorimotor coupling with multipart music. Journal of Experimental Psychology. Human Perception and Performance, 40(4), 1679–1696. 10.1037/a0037154 0096-1523 24979362

[b26] Iversen, J. R., Repp, B. H., & Patel, A. D. (2009). Top-down control of rhythm perception modulates early auditory responses. Annals of the New York Academy of Sciences, 1169(1), 58–73. 10.1111/j.1749-6632.2009.04579.x 0077-8923 19673755

[b71] Janata, P. (2009). The neural architecture of music-evoked autobiographical memories. Cerebral Cortex (New York, N.Y.), 19(11), 2579–2594. 10.1093/cercor/bhp008 1047-3211 19240137 PMC2758676

[b97] Janata, P. (2012). Acuity of mental representations of pitch. Annals of the New York Academy of Sciences, 1252(1), 214–221. 10.1111/j.1749-6632.2011.06441.x 0077-8923 22524362

[b59] Janata, P., Tomic, S. T., & Haberman, J. M. (2012). Sensorimotor coupling in music and the psychology of the groove. Journal of Experimental Psychology. General, 141(1), 54–75. 10.1037/a0024208 0096-3445 21767048

[b82] Johnson, P. C. D. (2014). Extension of Nakagawa & Schielzeth’s *R*^2^_GLMM_ to random slopes models. Methods in Ecology and Evolution, 5(9), 944–946. 10.1111/2041-210X.12225 2041-210X 25810896 PMC4368045

[b4] Jones, M. R., Johnston, H. M., & Puente, J. (2006). Effects of auditory pattern structure on anticipatory and reactive attending. Cognitive Psychology, 53(1), 59–96. 10.1016/j.cogpsych.2006.01.003 0010-0285 16563367

[b13] Jones, M. R., & Boltz, M. (1989). Dynamic attending and responses to time. Psychological Review, 96(3), 459–491. 10.1037/0033-295X.96.3.459 0033-295X 2756068

[b44] Joshi, S., Li, Y., Kalwani, R. M., & Gold, J. I. (2016). Relationships between Pupil Diameter and Neuronal Activity in the Locus Coeruleus, Colliculi, and Cingulate Cortex. Neuron, 89(1), 221–234. 10.1016/j.neuron.2015.11.028 0896-6273 26711118 PMC4707070

[b55] Kang, O., & Wheatley, T. (2015). Pupil dilation patterns reflect the contents of consciousness. Consciousness and Cognition, 35, 128–135. 10.1016/j.concog.2015.05.001 1053-8100 26002764

[b67] King-Smith, P. E., Grigsby, S. S., Vingrys, A. J., Benes, S. C., & Supowit, A. (1994). Efficient and unbiased modifications of the QUEST threshold method: Theory, simulations, experimental evaluation and practical implementation. Vision Research, 34(7), 885–912. 10.1016/0042-6989(94)90039-6 0042-6989 8160402

[b53] Laeng, B., Eidet, L. M., Sulutvedt, U., & Panksepp, J. (2016). Music chills: The eye pupil as a mirror to music’s soul. Consciousness and Cognition, 44, 161–178. 10.1016/j.concog.2016.07.009 1053-8100 27500655

[b91] Laeng, B., Sirois, S., & Gredebäck, G. (2012). Pupillometry: A Window to the Preconscious? Perspectives on Psychological Science, 7(1), 18–27. 10.1177/1745691611427305 1745-6916 26168419

[b17] Lakatos P, Karmos G, Mehta AD, Ulbert I, Schroeder CE Entrainment of neuronal oscillations as a mechanism of attentional selection. Science (80-) . 2008;320(5872):110–3. 10.1126/science.1154735 18388295

[b48] Lange, E. B., Zweck, F., & Sinn, P. (2017). Microsaccade-rate indicates absorption by music listening. Consciousness and Cognition, 55, 59–78. 10.1016/j.concog.2017.07.009 1053-8100 28787663

[b27] Large, E. W., Herrera, J. A., & Velasco, M. J. (2015). Neural Networks for Beat Perception in Musical Rhythm. Frontiers in Systems Neuroscience, 9, 159. 10.3389/fnsys.2015.00159 1662-5137 26635549 PMC4658578

[b15] Large, E. W., & Palmer, C. (2002). Perceiving temporal regularity in music. Cognitive Science, 26(1), 1–37. 10.1207/s15516709cog2601_1 0364-0213

[b14] Large, E. W., & Snyder, J. S. (2009). Pulse and meter as neural resonance. Annals of the New York Academy of Sciences, 1169(1), 46–57. 10.1111/j.1749-6632.2009.04550.x 0077-8923 19673754

[b61] Leman, M., Lesaffre, M., & Tanghe, K. (2001). Computer code IPEM Toolbox. Ghent, Belgium: Ghent University.

[b51] Liao, H. I., Yoneya, M., Kidani, S., Kashino, M., & Furukawa, S. (2016). Human Pupillary Dilation Response to Deviant Auditory Stimuli: Effects of Stimulus Properties and Voluntary Attention. Frontiers in Neuroscience, 10, 43. 10.3389/fnins.2016.00043 1662-4548 26924959 PMC4756168

[b1] London J. Hearing in Time (2012). Psychological Aspects of Musical Meter. New York, NY: Oxford University Press.

[b31] Maróti, E., Knakker, B., Vidnyánszky, Z., & Weiss, B. (2017). The effect of beat frequency on eye movements during free viewing. Vision Research, 131, 57–66. 10.1016/j.visres.2016.12.009 0042-6989 28057578

[b68] Marvit, P., Florentine, M., & Buus, S. (2003). A comparison of psychophysical procedures for level-discrimination thresholds. The Journal of the Acoustical Society of America, 113(6), 3348–3361. 10.1121/1.1570445 0001-4966 12822806

[b90] Mathôt, S. (2018). Pupillometry: Psychology, Physiology, and Function. Journal of Cognition, 1(1), 16. 10.5334/joc.18 2514-4820 31517190 PMC6634360

[b89] Mathôt, S., & Van der Stigchel, S. (2015). New Light on the Mind’s Eye: The Pupillary Light Response as Active Vision. Current Directions in Psychological Science, 24(5), 374–378. 10.1177/0963721415593725 0963-7214 26494950 PMC4601080

[b70] MATLAB. (2017). Natick, MA: MathWorks, Inc.

[b62] McCloy, D. R., Larson, E. D., Lau, B., & Lee, A. K. C. (2016). Temporal alignment of pupillary response with stimulus events via deconvolution. The Journal of the Acoustical Society of America, 139(3), EL57–EL62. 10.1121/1.4943787 0001-4966 27036288 PMC5392052

[b42] McGinley, M. J., David, S. V., & McCormick, D. A. (2015). Cortical Membrane Potential Signature of Optimal States for Sensory Signal Detection. Neuron, 87(1), 179–192. 10.1016/j.neuron.2015.05.038 0896-6273 26074005 PMC4631312

[b12] Miller, J. E., Carlson, L. A., & McAuley, J. D. (2013). When what you hear influences when you see: Listening to an auditory rhythm influences the temporal allocation of visual attention. Psychological Science, 24(1), 11–18. 10.1177/0956797612446707 0956-7976 23160202

[b22] Morillon, B., Hackett, T. A., Kajikawa, Y., & Schroeder, C. E. (2015). Predictive motor control of sensory dynamics in auditory active sensing. Current Opinion in Neurobiology, 31, 230–238. 10.1016/j.conb.2014.12.005 0959-4388 25594376 PMC4898262

[b28] Morillon, B., Schroeder, C. E., & Wyart, V. (2014). Motor contributions to the temporal precision of auditory attention. Nature Communications, 5(1), 5255. 10.1038/ncomms6255 2041-1723 25314898 PMC4199392

[b43] Murphy, P. R., Robertson, I. H., Balsters, J. H., & O’connell, R. G. (2011). Pupillometry and P3 index the locus coeruleus-noradrenergic arousal function in humans. Psychophysiology, 48(11), 1532–1543. 10.1111/j.1469-8986.2011.01226.x 0048-5772 21762458

[b45] Murphy, P. R., O’Connell, R. G., O’Sullivan, M., Robertson, I. H., & Balsters, J. H. (2014). Pupil diameter covaries with BOLD activity in human locus coeruleus. Human Brain Mapping, 35(8), 4140–4154. 10.1002/hbm.22466 1065-9471 24510607 PMC6869043

[b94] Näätänen, R., Paavilainen, P., Rinne, T., & Alho, K. (2007). The mismatch negativity (MMN) in basic research of central auditory processing: A review. Clinical Neurophysiology, 118(12), 2544–2590. 10.1016/j.clinph.2007.04.026 1388-2457 17931964

[b39] Naber, M., Alvarez, G. A., & Nakayama, K. (2013). Tracking the allocation of attention using human pupillary oscillations. Frontiers in Psychology, 4, 919. 10.3389/fpsyg.2013.00919 1664-1078 24368904 PMC3857913

[b83] Nakagawa, S., Johnson, P. C. D., & Schielzeth, H. (2017). The coefficient of determination *R*^2^ and intra-class correlation coefficient from generalized linear mixed-effects models revisited and expanded. Journal of the Royal Society, Interface, 14(134), 20170213. 10.1098/rsif.2017.0213 1742-5689 28904005 PMC5636267

[b84] Nakagawa, S., & Schielzeth, H. (2013). A general and simple method for obtaining R2 from Generalized Linear Mixed-effects Models. Methods in Ecology and Evolution, 4(2), 133–142. 10.1111/j.2041-210x.2012.00261.x 2041-210X

[b96] Navarro Cebrian, A., & Janata, P. (2010). Electrophysiological correlates of accurate mental image formation in auditory perception and imagery tasks. Brain Research, 1342, 39–54. 10.1016/j.brainres.2010.04.026 0006-8993 20406623

[b93] Nieuwenhuis, S., De Geus, E. J., & Aston-Jones, G. (2011). The anatomical and functional relationship between the P3 and autonomic components of the orienting response. Psychophysiology, 48(2), 162–175. 10.1111/j.1469-8986.2010.01057.x 0048-5772 20557480 PMC3797154

[b24] Nozaradan, S., Peretz, I., & Keller, P. E. (2016). Individual Differences in Rhythmic Cortical Entrainment Correlate with Predictive Behavior in Sensorimotor Synchronization. Scientific Reports, 6(1), 20612. 10.1038/srep20612 2045-2322 26847160 PMC4742877

[b65] Nozaradan, S., Peretz, I., & Mouraux, A. (2012). Selective neuronal entrainment to the beat and meter embedded in a musical rhythm. The Journal of Neuroscience : The Official Journal of the Society for Neuroscience, 32(49), 17572–17581. 10.1523/JNEUROSCI.3203-12.2012 0270-6474 23223281 PMC6621650

[b74] Pinheiro J, Bates D, DebRoy S, Sarkar D, Team the RDC. nlme: Linear and nonlinear mixed effects models (Version 3.1-113). . 2013.

[b92] Polich, J. (2007). Updating P300: An integrative theory of P3a and P3b. Clinical Neurophysiology, 118(10), 2128–2148. 10.1016/j.clinph.2007.04.019 1388-2457 17573239 PMC2715154

[b75] R Core Team (2013). R: A language and environment for statistical computing. R Foundation for Statistical Computing, Vienna, Austria. Retrieved from http://www.R-project.org/

[b37] Rajkowski, J., Kubiak, P., & Aston-Jones, G. (1993). Correlations between locus coeruleus (LC) neural activity, pupil diameter, and behavior in monkey support a role of LC in attention. Abstracts - Society for Neuroscience, 19(974).0190-5295

[b32] Recanzone, G. H. (2002). Auditory influences on visual temporal rate perception. Journal of Neurophysiology, 89(2), 1078–1093. 10.1152/jn.00706.2002 0022-3077 12574482

[b23] Repp, B. H. (2005). Sensorimotor synchronization: A review of the tapping literature. Psychonomic Bulletin & Review, 12(6), 969–992. 10.3758/BF03206433 1069-9384 16615317

[b86] Robin, X., Turck, N., Hainard, A., Tiberti, N., Lisacek, F., Sanchez, J. C., & Müller, M. (2011). pROC: An open-source package for R and S+ to analyze and compare ROC curves. BMC Bioinformatics, 12(1), 77. 10.1186/1471-2105-12-77 1471-2105 21414208 PMC3068975

[b69] S.R. Research Eyelink 1000 User Manual. Ltd. SRR, editor. Vol. 1.5.0. Ontario, Canada: SR Research Ltd.; 2009.

[b38] Sara, S. J. (2015). Locus Coeruleus in time with the making of memories. Current Opinion in Neurobiology, 35, 87–94. 10.1016/j.conb.2015.07.004 0959-4388 26241632

[b30] Schaefer, K. P., Süss, K. J., & Fiebig, E. (1981). Acoustic-induced eye movements. Annals of the New York Academy of Sciences, 374(1 Vestibular an), 674–688. 10.1111/j.1749-6632.1981.tb30910.x 0077-8923 6951453

[b16] Schroeder, C. E., & Lakatos, P. (2009). Low-frequency neuronal oscillations as instruments of sensory selection. Trends in Neurosciences, 32(1), 9–18. 10.1016/j.tins.2008.09.012 0166-2236 19012975 PMC2990947

[b18] Schroeder, C. E., Wilson, D. A., Radman, T., Scharfman, H., & Lakatos, P. (2010). Dynamics of Active Sensing and perceptual selection. Current Opinion in Neurobiology, 20(2), 172–176. 10.1016/j.conb.2010.02.010 0959-4388 20307966 PMC2963579

[b80] Schwarz, G. (1978). Estimating the dimension of a model. Annals of Statistics, 6(2), 461–464. 10.1214/aos/1176344136 0090-5364

[b33] Sekuler, R., Sekuler, A. B., & Lau, R. (1997). Sound alters visual motion perception. Nature, 385(6614), 308. 10.1038/385308a0 0028-0836 9002513

[b77] Selya, A. S., Rose, J. S., Dierker, L. C., Hedeker, D., & Mermelstein, R. J. (2012). A practical guide to calculating Cohen’s f2, a measure of local effect size, from PROC MIXED. Frontiers in Psychology, 3, 111. 10.3389/fpsyg.2012.00111 1664-1078 22529829 PMC3328081

[b20] Siegel, M., Donner, T. H., & Engel, A. K. (2012). Spectral fingerprints of large-scale neuronal interactions. Nature Reviews. Neuroscience, 13(2), 121–134. 10.1038/nrn3137 1471-003X 22233726

[b29] Teki, S., Grube, M., Kumar, S., & Griffiths, T. D. (2011). Distinct neural substrates of duration-based and beat-based auditory timing. The Journal of Neuroscience : The Official Journal of the Society for Neuroscience, 31(10), 3805–3812. 10.1523/JNEUROSCI.5561-10.2011 0270-6474 21389235 PMC3074096

[b66] Tomic, S. T., & Janata, P. (2007). Ensemble: A web-based system for psychology survey and experiment management. Behavior Research Methods, 39(3), 635–650. 10.3758/BF03193036 1554-351X 17958178

[b57] Tomic, S. T., & Janata, P. (2008). Beyond the beat: Modeling metric structure in music and performance. The Journal of the Acoustical Society of America, 124(6), 4024–4041. 10.1121/1.3006382 0001-4966 19206825

[b41] Trost, J. W., Labbé, C., & Grandjean, D. (2017). Rhythmic entrainment as a musical affect induction mechanism. Neuropsychologia, 96, 96–110. 10.1016/j.neuropsychologia.2017.01.004 0028-3932 28069444

[b72] Wang, C. A., Boehnke, S. E., Itti, L., & Munoz, D. P. (2014). Transient pupil response is modulated by contrast-based saliency. The Journal of Neuroscience : The Official Journal of the Society for Neuroscience, 34(2), 408–417. 10.1523/JNEUROSCI.3550-13.2014 0270-6474 24403141 PMC6608151

[b47] Weiss, M. W., Trehub, S. E., Schellenberg, E. G., & Habashi, P. (2016). Pupils dilate for vocal or familiar music. Journal of Experimental Psychology. Human Perception and Performance, 42(8), 1061–1065. 10.1037/xhp0000226 0096-1523 27123682

[b73] Welch PD The Use of the Fast Fourier Transform for the Estimation of Power Spectra: A Method Based on Time Averaging Over Short, Modified Periodograms. IEEE® Trans Audio Electroacoust. 1967;AU-15:70–3.

[b64] Wierda, S. M., van Rijn, H., Taatgen, N. A., & Martens, S. (2012). Pupil dilation deconvolution reveals the dynamics of attention at high temporal resolution. Proceedings of the National Academy of Sciences of the United States of America, 109(22), 8456–8460. 10.1073/pnas.1201858109 0027-8424 22586101 PMC3365158

[b3] Yee, W., Holleran, S., & Jones, M. R. (1994). Sensitivity to event timing in regular and irregular sequences: Influences of musical skill. Perception & Psychophysics, 56(4), 461–471. 10.3758/BF03206737 0031-5117 7984401

